# cAMP Bursts Control T Cell Directionality by Actomyosin Cytoskeleton Remodeling

**DOI:** 10.3389/fcell.2021.633099

**Published:** 2021-05-20

**Authors:** Morgane Simao, Fabienne Régnier, Sarah Taheraly, Achille Fraisse, Rachida Tacine, Marie Fraudeau, Adam Benabid, Vincent Feuillet, Mireille Lambert, Jérôme Delon, Clotilde Randriamampita

**Affiliations:** ^1^Université de Paris, Institut Cochin, INSERM, CNRS, Paris, France; ^2^Master de Biologie, École Normale Supérieure de Lyon, Université Claude Bernard Lyon I, Université de Lyon, Lyon, France

**Keywords:** lymphocyte, migration, cAMP, actomyosin, chemokine

## Abstract

T lymphocyte migration is an essential step to mounting an efficient immune response. The rapid and random motility of these cells which favors their sentinel role is conditioned by chemokines as well as by the physical environment. Morphological changes, underlaid by dynamic actin cytoskeleton remodeling, are observed throughout migration but especially when the cell modifies its trajectory. However, the signaling cascade regulating the directional changes remains largely unknown. Using dynamic cell imaging, we investigated in this paper the signaling pathways involved in T cell directionality. We monitored cyclic adenosine 3′-5′ monosphosphate (cAMP) variation concomitantly with actomyosin distribution upon T lymphocyte migration and highlighted the fact that spontaneous bursts in cAMP starting from the leading edge, are sufficient to promote actomyosin redistribution triggering trajectory modification. Although cAMP is commonly considered as an immunosuppressive factor, our results suggest that, when transient, it rather favors the exploratory behavior of T cells.

## Introduction

Fast and random motility of T lymphocytes is a prerequisite to perform efficient immune surveillance, as these cells need to scan the widest possible area in a short time within secondary lymphoid organs (Krummel et al., 2016). This motility is conditioned by the chemical (mainly chemokines) and physical (structural constraints) parameters specifically found in these confined environments. Even in the absence of physical obstacles, random migration is observed (see (Real et al., 2004) for instance), suggesting the existence of cell-intrinsic factors regulating the cell directionality.

T cells stimulated by chemokines lose their round shape within a few minutes, to acquire a clear polarized shape with a front, the lamellipodium, and a rear, the uropod. This asymmetry, required for their migration, is achieved by a rapid modification of their cytoskeleton (Moreau et al., 2018). In fact, chemokine stimulation triggers a rapid increase in polymerized actin (Real et al., 2007), especially branched actin which accumulates at the cell front giving the lamellipodium some highly dynamic properties adapted to the research strategy of T lymphocytes. Conversely, stable actin and actomyosin allow to maintain the structural shape of the cell body and to promote its contractility (Dupré et al., 2015; Chugh and Paluch, 2018). During T cell migration, continuous remodeling of the cytoskeleton, such as the actin network, has to take place, especially each time cells modify their trajectory. Indeed, in this case, the cell slows down and its lamellipodium retracts, leading to the transient loss of cell asymmetry before being reestablished along another axis. Calcium has been clearly identified as the stop signal leading to lamellipodium retraction and migration inhibition when T cells encounter an antigen-presenting cell (Donnadieu et al., 1994; Dong et al., 2017). However, the signaling pathway involved in shape changes of chemokine-stimulated T cells during trajectory changes, remains unclear.

The role of cAMP upon migration remains confused depending on the cell types or the experimental conditions. In T lymphocytes, a negative effect of cAMP pathway has been known for a long time and is supported by different studies showing that agents inducing large increases in cAMP levels, such as forskolin, inhibitors of phosphodiesterases or prostaglandin E2, promote cell rounding and migration inhibition (Valitutti et al., 1993; Oppenheimer-Marks et al., 1994; Layseca-Espinosa et al., 2003; Dong et al., 2006). Interestingly, in other cell types, cAMP seems to play a more complex role in cell migration through its compartmentalization. In fibroblasts or epithelial cells, an increase of cAMP-activated protein kinase (PKA) activity at the leading edge has been reported to promote cell migration (Howe et al., 2005; Lim et al., 2008; McKenzie et al., 2020). Conversely, in neutrophils, local increases in cAMP promote uropod retraction through the regulation of the non-muscle myosin II by PKA (Liu et al., 2010). The development of powerful biosensors makes it possible to measure cAMP (Klarenbeek et al., 2011) at the subcellular level even in small cells such as lymphocytes and with a good temporal resolution, and therefore to revisit the role of cAMP in T cell migration.

Using dynamic cell imaging, we here investigate the signaling pathways involved in trajectory control during T cell migration. We demonstrate that transient spontaneous increases in intracellular cAMP are sufficient to drive T cell actomyosin reorganization, leading to paths modifications.

## Materials and Methods

### Cells

CEM T cells were cultured in RPMI 1640, supplemented with 10% FCS, 2 mM L-Glutamine, 50 U/ml penicillin and 50 μg/ml streptomycin. When specified, cells were transfected by nucleofection (Amaxa Nucleofactor, Lonza) with 5 μg DNA for 5 millions of cells using the C-016 program. The cells were used the day after nucleofection.

### Reagents

CXCL12 (recombinant human SDF1-α) was purchased from Peprotech (300-28A) and VCAM-1 (CD106 Fc chimera protein) from R&D Systems (862-VC-100). Calcium measurements were performed with Fura-2/AM (Molecular Probes, F1225). DMACM-caged 8-Br-cAMP was purchased from Biolog (D044). Nucleus labeling was performed with Hoechst (Molecular Probes, H1399). F-actin detection was performed by expressing the LifeAct-mCherry construct (gift from Dr. A. Benmerah). Myosin IIA was followed by expressing tagged form of Myosin Heavy Chain 9 (MyH9-GFP, gift from PJ Saez). Stable actin detection was performed with SiRActin (TebuBio, SC001) or with mRFP-Utrophin-CH (Addgene #64358).

### Live Imaging Acquisition

For migration experiments, glass coverslips were coated with 1 μg/ml CXCL12 and 1 μg/ml VCAM-1 overnight at 4°C. After rinsing, coverslips were kept in mammalian saline buffer (140 mM NaCl, 5 mM KCl, 1 mM CaCl_2_, 1 mM MgCl_2_, 20 mM HEPES, and 11 mM glucose) supplemented with 5% FCS. Cells were deposited on coverslips just before image acquisition started. Live imaging experiments were performed at 37°C with a wide-field Nikon TE2000, equipped with a CMOS camera (ORCA-flash4.0 LT, Hamamatsu). Images were acquired every 10 s with Metafluor software.

#### Actin, Myosin, and Nucleus

For total polymerized actin detection, cells were transfected with LifeAct-mCherry construct. For stable actin detection, cells were transfected with Utrophin-CH-RFP. For non-muscle Myosin IIA detection, cells were transfected with MyH9-GFP construct. For stable actin labeling, cells were incubated for 1 h with 250 nM SiRActin in complete medium at 37°C. After rinsing, cells were deposited on coated coverslips. Nucleus labeling was performed with 4 min incubation, using 2 μg/ml Hoechst. Distribution of compounds was followed by 650 nm Excitation/700 nm Emission for SiRActin, 560 nm Excitation/645 nm Emission for LifeAct-mCherry, 485 nm Excitation/525 nm Emission for MyH9-GFP and 360 nm Excitation/440 nm Emission for Hoechst.

#### cAMP Measurements

For cAMP measurements, cells were transfected with the most sensitive version of TEpacVV [H187 (Klarenbeek et al., 2015)]. TEpacVV was a gift from Dr. K. Jalink (Netherlands Cancer Institute). Experiments were performed 24 h after transfection, as previously described (Conche et al., 2009). Briefly, when cAMP increases, the probe undergoes a conformational change that allows a decrease of energy transfer between a turquoise molecule (Excitation 436 nm, Emission 470 nm) and two Venus molecules (Excitation 500 nm, Emission 535 nm) (Klarenbeek et al., 2011); the energy transfer can be measured as a change in FRET (Excitation 436 nm, Emission 535 nm). Three images were acquired every 10 s: visible, Turquoise channel and FRET channel. The ratio R = Turquoise/FRET, which gives an estimate of cAMP concentration, was calculated with MetaFluor (Roper Scientific) after background subtraction. An increase of this ratio corresponds to an increase in cAMP concentration.

#### Calcium Measurements

For calcium experiments, cells were loaded with 500 nM Fura-2/AM for 20 min at 37°C. Excitation was performed alternatively at 350 and 380 nm and emission recorded at 510 nm. The ratio (Exc 350, Em510/Exc380, Em510) was calculated with MetaFluor (Roper Scientific) after background subtraction. For combined cAMP and Ca measurements, TEpacVV-transfected cells were loaded only with 200 nM Fura-2/AM in order to minimize the crosstalk between the four fluorescence signals.

### Image Analysis

#### Cell Roundness

This parameter was quantified with ImageJ software and corresponds to: $\frac{4\times a\,r\,e\,a}{\pi \times {\left(m\,a\,j\,o\,r\,a\,x\,i\,s\right)}^{2}}$. It is equal to 1 for a round cell and < 1 for a polarized one.

#### Front/Back Ratios

With Metamorph software, a 6–9 pixels wide scanline along the cell axis was drawn. The front/back ratio was then calculated by dividing the intensity at the front edge to the one measured at the back one.

#### Angles

Angle measurements were performed with ImageJ by drawing lines along the polarization axes observed between two consecutive lamellipodia formation.

#### Pearson Coefficient

In cells transfected with MyH9-GFP and labeled with SiRActin, Pearson Coefficient was measured on Fiji (ImageJ software, version 1.51 u) by using a macro containing the Coloc2 plugin. This coefficient measures the degree of overlap between two stainings and was used to quantify the degree of colocalization between MyH9-GFP and SiRActin staining. A Pearson Coefficient value of 0 means that there is no colocalization between the two stainings. By contrast, a Pearson Coefficient value of 1 means that there is a perfect colocalization between MyH9-GFP and SiRActin.

#### Kymographs

Kymographs have been performed with Metamorph software by drawing a line as wide as the cell along the migration axis.

#### Cross-Correlation

Cross-correlation was used to study the correlation between cAMP variations and cell shape (roundness). The Pearson correlation coefficient (ρ) between two time courses was computed as a function of time lag (τ):

$$\rho \,\left(\tau \right)=\frac{\sum \left[\left(x\,\left(i\right)-\bar{x}\right)\times \left(y\,\left(i\mp \tau \right)-\bar{y}\right)\right]}{\sqrt{\sum {\left(x\,\left(i\right)-\bar{x}\right)}^{2}\times \sum {\left(y\,\left(i\mp \tau \right)-\bar{y}\right)}^{2}}};$$

$$w\,i\,t\,h\,x\,a\,n\,d\,y\,v\,a\,r\,i\,a\,b\,l\,e\,s\,a\,n\,d\,\bar{x}\,a\,n\,d\,\bar{y}\,v\,a\,r\,i\,a\,b\,l\,e\,m\,e\,a\,n\,s.$$

### Local Leading Edge Release of cAMP

CEM were incubated with 20 μM DMACM-caged 8-Br-cAMP for 3 h at 37°C. SiRActin (250 nM) was added to the medium during the last hour. Cells were rinsed and deposited on VCAM-1/CXCL12 coated coverslips for 30 min at 37°C. The experiment was performed by using an iMIC TILL Photonics microscope equipped with two cameras EMCCD (ANDOR Technology) and 60x objective (numerical aperture: 1.49) + 1.5 zoom. Images were acquired every 5 s. After four image acquisitions, the release of DMACM-caged 8-Br-cAMP was performed with a 405 nm laser (Toptica iBAEM 110 mV, 1 ms illumination, 100% power) by adjusting a 7 μm diameter region at the level of the leading edge of a migrating cell. For control experiments, a similar protocol was followed except that the cells were incubated with DMSO instead of caged-cAMP for the same duration.

### Traction Force Microscopy

Traction force microscopy experiments were performed with the help of Cell Biomechanics facility of Cochin Institute.

#### Hydrogel Preparation

Hydrogels (∼700Pa) were prepared with acrylamide (3%, Sigma #A4058), bis-acrylamide (0.3%, Sigma #M1533-25), streptavidin-acrylamide (Invitrogen, S21379) and Flash Red 0.2 μm fluorescent beads (Bangs Laboratories, FSFR002). Streptavidin-acrylamide was used at 1/100,000 molecular ratio to acrylamide as previously described (Saitakis et al., 2017). After activation with TEMED and ammonium persulfate, 11 μl of the polymerization mix was added on a non-functionalized 12 mm diameter coverslip. A functionalized glass coverslip coated with silane (Sigma, 17-1330-01) was placed on top. Polymerization was performed at room temperature for 30 min.

#### Mechanical Properties of the Polyacrylamide Gels

Gels were unmolded by removing the non-functionalized coverslip. We then checked whether the bead distribution on the top surface was suitable for traction forces measurement (∼2000 beads per 512 × 512 pixels field). The Young Modulus was then calculated according to (Gross and Kress, 2017). In brief, tungsten carbide spheres with known radius (0.4 and 0.6 mm) and density (15,630 g/l) were deposited on the hydrogel surface. We then measured the gel deformation induced by the bead by acquiring z-images of the fluorescent beads embedded in the gel, focusing on the bottom and the top of the gel with an indentation of 0.2 μm. By using ImageJ, we measured the gel height and the collapse distance of the sphere. The Young Modulus was calculated by using a R code based on (Gross and Kress, 2017) (available on demand).

#### Functionalization of Hydrogel Surface

We used the specific biotin-streptavidin binding and anti-Fc/Fc binding to form a sandwich of macromolecules for the functionalization of polyacrylamide gels. All gel surfaces were incubated with 10 μg/ml of a goat anti-human IgG Fc biotinylated antibody (Abcam: ab97223) in PBS-BSA 0.2% overnight at 4°C. Gels were then incubated with 10 μg/ml recombinant human VCAM-1/CD106 Fc chimera in PBS-BSA 0.2% for 2 h at 37°C. We were not able to experimentally assess the VCAM-1 surface density, but theoretically calculated the density of streptavidin molecules on the gel. For this we used the three assumptions enunciated in Saitakis et al. (2017). In brief, (1) the volume of the hydrated gel (with culture medium) that we were able to calculate with the thickness and the coverslip diameter, is approximately 40% bigger than the initial volume of the polymerization mix (Hynd et al., 2007). (2) All the streptavidin-acrylamide molecules within the polymerization mix polymerized within the gel. (3) Biotinylated anti-Fc antibody can access the first 10 nm of the gel (10 nm is the approximated size of the streptavidin molecule) due to their own size and the size of the pore reported in the literature (Trappmann et al., 2012). We then calculated that the theoretical surface density of streptavidin-acrylamide is 25 molecules/μm^2^.

#### Traction Force Measurements

Traction force microscopy experiments were performed with 20x objective (numerical aperture 0.75) and 1.5 zoom. CXCL12-stimulated (100 ng/ml) cells were deposited on a VCAM-1-coated gel for 30 min at 37°C. Transmitted light and corresponding fluorescent images of beads and actin were acquired every 10 s using the MetaMorph software.

#### Force Image Analysis

We first aligned images of the fluorescent beads to correct the drift by using the ImageJ plugin Stack Reg. The forces were calculated by the method described in Martiel et al. (2015). Basically, the displacement field was calculated by Particle Image Velocimetry (PIV) plugin implemented in ImageJ. The PIV was performed through an iterative process. For each iteration, the displacement was calculated by the normalized correlation coefficient algorithm, so that an individual interrogation window was compared with a larger searching window. Each subsequent iteration took into account the displacement field measured previously. The resulting final grid size for the displacement field was 5.04 μm × 5.04 μm with more than six beads per interrogation window on average. With the displacement field obtained by PIV analysis, the traction force field was reconstructed by the Fourier transform traction cytometry (FTTC) method (Martiel et al., 2015) with FTTC ImageJ plugin. The regularization parameter was set at 8 × 10^–11^ for all traction force reconstructions.

**FIGURE 1:**
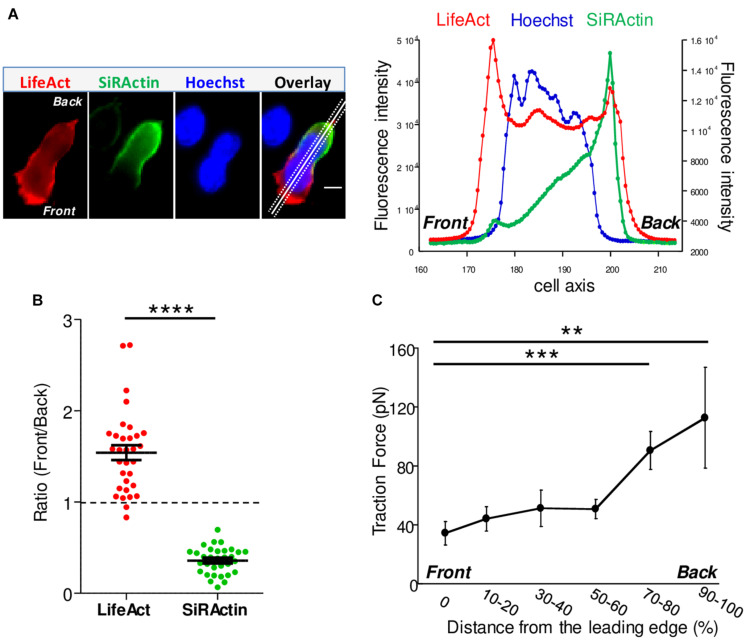
Actin cytoskeleton asymmetry upon chemokine-stimulated T cells. **(A)** T cells were transfected with LifeAct-mCherry and labeled with SiRActin and Hoechst. After washing, the cells were deposited on a VCAM-1/CXCL12 coated coverslip. A typical distribution of total polymerized actin (LifeAct), stable actin network (SiRActin) and nucleus (Hoechst) is shown on the left panel and quantified along the scanline displayed on the overlay image (right panel). Images are from Movie 2. Scale bar = 10 μm. **(B)** Using similar scanlines as in A, the ratio between front and rear intensities was measured for total polymerized actin and stable actin networks. Mean ± SE (*N* = 31). The values obtained were statistically different (paired *t*-test, ****p < 0.0001, *N* = 32 cells). **(C)** The subcellular distribution of forces was measured in CXCL12-stimulated T cells upon migration on approximately 700 Pa gels coated with VCAM-1. The magnitude of these forces was significantly higher at the back of the cells compared to the front. The values of the forces expressed in picoNewton correspond to the mean of forces ± SE measured in 14 different cells. ***p < 0.001, **p < 0.01 Kruskal-Wallis test.

**FIGURE 2:**
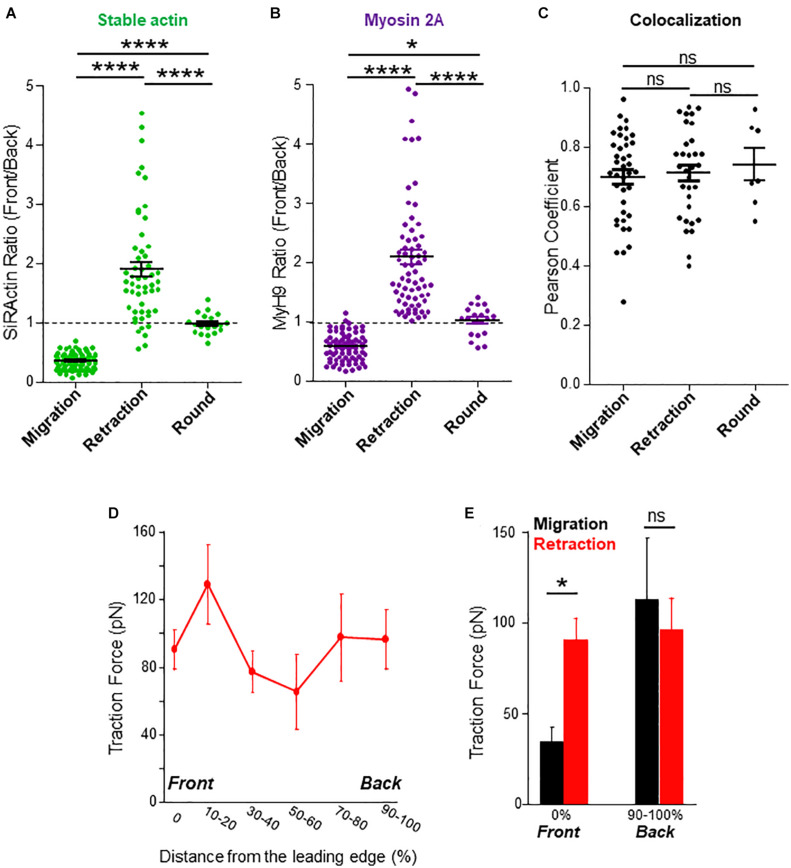
Stable actin relocalization. **(A)** The front to back ratio of SiRActin intensities were measured by drawing a scanline along the cell axis of cells upon migration, while the lamellipodium retracted and once the cell had rounded up. Values correspond to the mean ± SE of 85 events from 60 different cells (migration), 53 events from 28 different cells (lamellipodium retraction) and 19 rounding events from 16 different cells (after retraction). Statistical analysis was performed through a one way ANOVA test with a Tukey post-test. ****p < 0.0001. **(B)** The front to back ratio of MyH9-GFP intensities were measured by drawing a scanline along the cell axis of cells upon migration, while the lamellipodium retracted and once the cell had rounded up. Values correspond to the mean ± SE of 77 events from 42 different cells (migration), 69 events from 36 different cells (lamellipodium retraction) and 20 rounding events from 17 different cells (after retraction). Statistical analysis was performed through a one way ANOVA test with a Tukey post-test. ****p < 0.0001, *p < 0.05. **(C)** In cells transfected with MyH9-GFP and labeled with SiRActin, Pearson coefficient was measured upon migration, while the lamellipodium retracted and once the cell had rounded up. Values correspond to the mean ± SE of 39 events from 24 different cells (migration), 32 events from 21 different cells (lamellipodium retraction) and seven rounding events from seven different cells (after retraction). Statistical analysis was performed through a one way ANOVA test with a Tukey post-test. ns: not significant. **(D)** Subcellular distribution of forces was measured in CXCL12-stimulated T lymphocytes upon lamellipodium retraction on approximately 700 Pa gels coated with VCAM-1. The values of the forces expressed in picoNewton correspond to the mean of forces ± SE measured in eight different cells retracting their lamellipodium. **(E)** Comparison of the values of the forces measured in 14 migrating cells (black) or in eight cells retracting their lamellipodium (red) at their front (0%) or their back (90–100%). *p < 0.05 Kruskal-Wallis test.

After this calculation, the forces along the cell body were isolated. The cell length was normalized by establishing that the cell front corresponds to 0% and the back to 100%. When specified (Figures 1C, 2D,E), the forces along the cell axis were pooled.

### Statistics

The statistical tests used for sample comparison are specified in the figure legends. In the figures, ns: not significant. They were performed with GraphPad software or RStudio.

## Results

### Remodeling of Actin Cytoskeleton in Chemokine-Stimulated T Lymphocytes

Upon chemokine stimulation, T cells lose their symmetrical shape and become polarized. This modification can be visualized by depositing CEM T cells, a lymphoblastic cell line which expresses CXCR4, the receptor of the CXCL12 chemokine (C-X-C motif chemokine 12 or stromal cell derived factor 1), on a glass coverslip coated with the integrin VCAM-1 (vascular cell adhesion molecule 1) and CXCL12. In these conditions, cells randomly migrate at a speed of 6.19 ± 0.03 μm/min (*N* = 80 cells) (illustrated in Supplementary Movie 1). In order to follow in real time and at the subcellular level cytoskeleton reorganization, T cells were transfected with mCherry-tagged LifeAct, a peptide able to bind to F-Actin. As shown in Figure 1A, polymerized actin is observed mainly at the front of the cell which corresponds to newly polymerized actin, as previously described (Real et al., 2007). In order to distinguish this pool from stable actin which constitutes the main pool of F-actin in unstimulated cells, SiRActin [a fluorescent cell-permeable F-Actin binding compound (Milroy et al., 2012; Lukinavicius et al., 2014)] was used. Resting cells were incubated for 1 h with SiRActin, rinsed and then stimulated so that the newly polymerized actin was not labeled. In these conditions, we clearly observed that, contrary to total polymerized actin, the stable actin network was restricted to the back of the cell behind the nucleus (Figure 1A and Supplementary Movie 2). The distribution of the two actin networks was quantified by drawing a scanline along the antero-posterior axis of the cell and then by measuring the ratio of intensities between the front and the back as shown in the example presented in Figure 1A. A ratio superior to 1 indicates an accumulation at the cell front. A statistical difference was measured between the localization of these two actin networks: total polymerized actin accumulates at the cell front while stable actin mainly accumulates at the back (Figure 1B). We next wondered whether the polarization of actin networks could be correlated with a mechanical asymmetry during T cell migration. To answer this question, we used the dynamic Traction Force Microscopy technique (Nerger et al., 2017) which allows the forces developed by the cells upon migration on polyacrylamide (PAA) gels to be measured. T lymphocytes are fast-moving cells, therefore they are expected to develop low forces on their substrate. For this reason, we used soft PAA gels of about 700 Pa and measured how T cells were able to displace fluorescent beads embedded within the PAA gel while they migrate. As quantified in Figure 1C and illustrated in Supplementary Figures 1A,C and T cells clearly imprint centripetal forces with a maximum intensity at the back of the cells and minimal intensity at the front, as previously observed in neutrophils (Jannat et al., 2011). The intensity of the forces we measured was very low, i.e., 100 times smaller than what was measured in neutrophils on gels with comparable stiffness (Jannat et al., 2011). We can therefore conclude that, during migration, T lymphocytes adhere mainly, but poorly, where stable actin accumulates.

### Actomyosin Relocalization Upon Trajectory Modification

The asymmetrical distribution of SiRActin remains stable upon migration. We thus investigated its behavior when cells retract their lamellipodium. This step is specifically required when cells round up and eventually change their direction. The example presented in Supplementary Figures 2A,B (kymograph and corresponding thumbnails) summarizes the different steps: upon migration, stable actin remains accumulated at the back of the cell (step 1) and (step 3); the retraction of the lamellipodium is accompanied by the relocalization of stable actin at the front where it rapidly accumulates (step 2) and (step 4). If the cell changes its direction (step 2), the stable actin will migrate entirely to this point which will constitute the new back of the migrating cell, as shown on the kymograph (Supplementary Figure 2A, red arrow). Conversely, if the cells round up (step 4) (yellow arrow), the stable actin will progressively redistribute homogeneously all around the cell membrane (step 5). The complete series of images is displayed in Supplementary Movie 3. The distribution of the stable actin is quantified on a series of cells during these different steps (Figure 2A): while, as previously shown in Figure 1B, it is clearly accumulated at the back upon migration (*R* = 0.35 ± 0.01, *N* = 85 events from 60 different cells), a transient accumulation at the front is observed during lamellipodium retraction (*R* = 1.89 ± 0.13, *N* = 53 events from 28 different cells) before it disperses around the membrane (*R* = 0.98 ± 0.04, *N* = 19 events from 16 different cells) when the cell rounds up. A similar relocalization of stable actin was observed when, having developed two lamellipodia, a cell retracts one of them (Supplementary Figure 2C). To confirm the results we obtained with SiRActin, we used another marker of stable actin, Utrophin-CH (Melak et al., 2017). In this case, a relocalization of stable actin network similar to the one detected with SiRActin is also observed upon lamellipodium retraction (Supplementary Figures 3A,B). Interestingly, non-muscle myosin-IIA [the main myosin isoform in T lymphocytes (Jacobelli et al., 2004)] displays a distribution similar to that of SiRActin as attested by Myosin Heavy Chain 9 (MyH9) localization upon migration, retraction and in round cells (Figure 2B). Indeed, MyH9 clearly accumulates at the cell back upon migration (*R* = 0.58 ± 0.03, *N* = 77 events from 42 different cells), relocalizes to the cell front during lamellipodium retraction (*R* = 2.09 ± 0.13, *N* = 69 events from 36 different cells) while it distributes uniformly when the cell rounds up (*R* = 1.02 ± 0.06, *N* = 20 events from 17 different cells). In these three configurations, a colocalization is observed between SiRActin and MyH9 distributions as shown in the example presented in Supplementary Figure 2B and Supplementary Movie 4. The strong correlation between the two markers is attested by Pearson Coefficient (PC) ≥ 0.7 (PC = 0.70 ± 0.02, *N* = 39 events from 24 migratory cells; PC = 0.71 ± 0.03, *N* = 32 events from 21 cells retracting their lamellipodium; PC = 0.74 ± 0.05, *N* = 7 events from 7 cells rounding up) (Figure 2C). This result suggests that the stable actin network detected by SiRActin mainly corresponds to actomyosin. Lamellipodium retractions were associated with a modification of the cell shape: the cell rounds up before it eventually elongates in another direction. We therefore measured simultaneously over time, the relocalization of SiRActin to the lamellipodium (measurement of the front/back ratio along the cell axis) together with the cell roundness and quantified the delay between the two events. We observed that the accumulation of actin at the front starts at 8.7 ± 4.0 s (*N* = 38 cells) before the cells begin to round up, suggesting that the relocalization of the stable actin might drive the retraction of the lamellipodium. Finally, we examined whether the relocalization of stable actin was accompanied by a redistribution of the forces developed by the cells. As shown in Figure 2D and illustrated in Supplementary Figures 1B,C, once again the distribution of high intensity forces is similar to that of stable actin: upon retraction, contrary to migratory conditions, centripetal forces at the level of the lamellipodium, reached intensities similar to those observed at the back of the cell. The intensities at the cell front were statistically higher than those observed in migrating cells, while no differences were observed at the back (Figure 2E). Once the cells have rounded, forces can no longer be measured (Supplementary Figure 1D).

### cAMP Variations Upon Trajectory Modification

**FIGURE 3:**
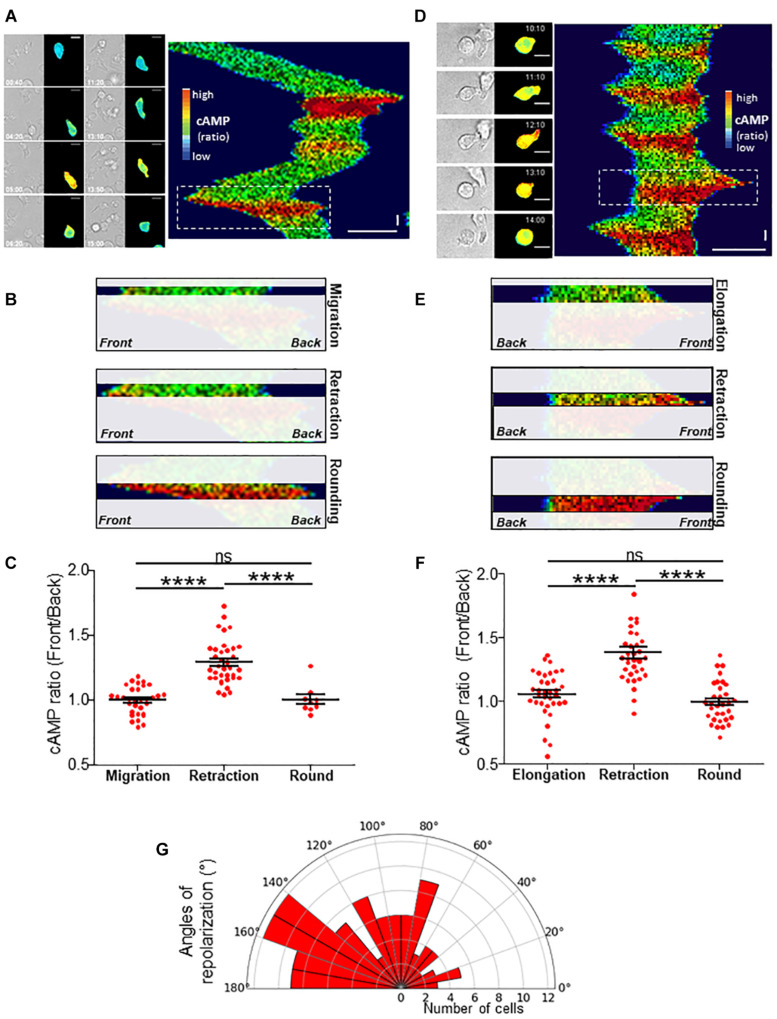
cAMP variations upon migration. **(A)** Example of a TEpacVV-transfected T cell migrating on a CXCL12/VCAM-1-coated coverslip. The corresponding kymograph was established along the antero-posterior axis of the cell. The x axis corresponds to the average cAMP level along the cell while the y axis corresponds to time. cAMP levels were coded in false colors. The complete series of images is shown in Supplementary Movie 5. Horizontal scale bar = 10 μm, vertical scale bar = 1 min. **(B)** Zoom of the zone corresponding to the white rectangle in the kymograph presented in **(A)** illustrating that the increase of cAMP starts from the front before invading the whole cell. **(C)** Ratios of cAMP level from the front to the back of the cell were measured by drawing scanlines along antero-posterior axis in migrating cells, cells retracting their lamellipodium or after they rounded up. Values correspond to the mean ± SE of 32 events (migration), 36 events (retraction), 8 values (round cells after retraction) from 11 different cells. Statistical analysis was performed through a one way ANOVA test with a Tukey post-test. ****p < 0.0001. **(D)** Example of cAMP variations measured in a TEpacVV-transfected T cell displaying elongation/retraction cycles on a CXCL12/VCAM-1-coated coverslip. During recording, the cell presents 5 such cycles as displayed on the kymograph. The complete series of images is displayed in Supplementary Movie 6. Horizontal scale bar = 10 μm, vertical scale bar = 1 min. **(E)** Zoom of the zone corresponding to the white rectangle in the kymograph presented in **(D)** illustrating that the increase of cAMP starts at the front before invading the whole cell. **(F)** Ratios of front to back cAMP levels measured in cells displaying elongation/retraction cycles. Values correspond to the mean ± SE of 35 values (elongation), 35 values (lamellipodium retraction) and 32 values (round cells after retraction) from 9 different cells. Statistical analysis was performed through a one way ANOVA test with a Tukey post-test. ****p < 0.0001.**(G)** Polar distribution of repolarization angles. Values correspond to the angle formed between polarization axes observed between two consecutive lamellipodia separated by cAMP-induced lamellipodium retraction. Width of sectors = 10°. Values correspond to 111 measurements performed on 37 different cells.

**FIGURE 4:**
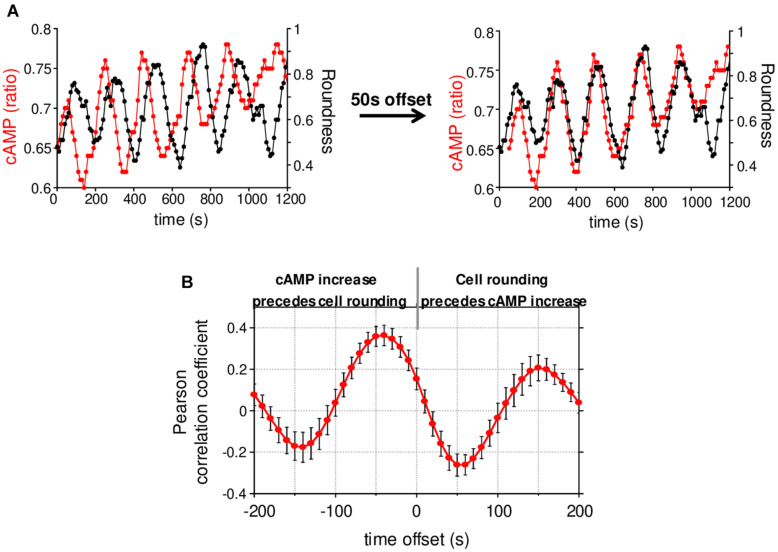
cAMP variations and cell roundness. **(A)** Example of cAMP variations measured simultaneously with the cell roundness for the cell presented in Figure 3D. The shift of cAMP curve by 50 s allowed us to synchronize it with the cell roundness curve. **(B)** Cross-correlation between cAMP and cell roundness. A negative offset means cAMP increase precedes the cell rounding. Values correspond to the mean ± SE of cross-correlation coefficients measured on 17 cells displaying cAMP oscillations.

We next wondered what the signaling pathway which triggers changes of direction and the simultaneous redistribution of the stable actin might be. Calcium has recently been associated with pausing upon confinement-induced T cell migration (Dong et al., 2017). Although, in our conditions, calcium (Ca) transients could sometimes be observed upon migration, they were neither systematic (Supplementary Figure 4A), nor associated with change of direction (Supplementary Figure 4B). We therefore focused on cAMP which has also been described as playing a role during migration (Howe, 2004). We used the very sensitive FRET biosensor, TEpacVV (Klarenbeek et al., 2011, 2015) to follow intracellular cAMP levels. As shown in the example presented in Figure 3A and Supplementary Movie 5, cAMP levels remained low upon migration, except at very specific moments when the cell stopped and eventually changed its direction. This can be visualized on the associated kymograph by the red zones corresponding to high cAMP levels. By zooming in on a change of direction (Figure 3A, white dotted rectangle), it appears that the cAMP increase starts at the cell front before invading the whole cell (Figure 3B). By combining cAMP and Ca measurements, we were able to demonstrate that no Ca variations could be detected in cells presenting some cAMP transients upon change of direction (Supplementary Figure 4C). The cellular heterogeneity in cAMP ratio was quantified by drawing scanlines along the antero-posterior axis of migrating cells. The front to back ratios were compared in cells which migrate, retract their lamellipodia or round up. While this ratio is equal to 1.00 ± 0.02 (*N* = 32 cells) upon migration, it increases up to 1.30 ± 0.03 (*N* = 32 cells) when the cells retract their lamellipodium before decreasing back to 1.00 ± 0.04 (*N* = 8 cells) once the cells have rounded up, meaning that lamellipodium retraction is associated with a local increase of cAMP at the cell front (Figure 3C). In our experimental conditions, some cells failed to migrate and went on repetitive elongation/retraction cycles (Figures 3D–F). Interestingly, these cells displayed cAMP oscillations (see Supplementary Figure 5A for two examples) with a very similar period from cell to cell (211.8 ± 11.7 s, *N* = 29 cells, Supplementary Figure 5B) and which is very regular for a given cell (Supplementary Figure 5C). These oscillations were associated with morphological changes corresponding to elongation/retraction cycles during which the level of cAMP starts to rise in the lamellipodium before invading the whole cell when it rounds up (Figure 3D and zoom in Figure 3E). The complete series of images is displayed in Supplementary Movie 6. In these cells, the antero-posterior ratio of cAMP was 1.05 ± 0.03 (*N* = 35 cells) upon elongation, increased to 1.38 ± 0.05 (*N* = 35 cells) upon retraction, before decreasing to 0.99 ± 0.03 (*N* = 32 cells) in round cells (Figure 3F). These values were very similar to those measured in migrating cells (Figure 3C). In order to quantify the coupling between cAMP level and the shape of the cells, the roundness was measured simultaneously with cAMP as in the example presented in Figure 4A (left panel). Clearly, the two parameters oscillate at the same frequency. However, an offset of 50 s is necessary in this example to synchronize cAMP levels and roundness (Figure 4A, right panel). A cross-correlation analysis was performed for a series of cells (see methods for details) and reveals that the most significant positive correlation between the two parameters (0.36 ± 0.05, *N* = 17 cells) is obtained with a 40–50 s temporal offset (Figure 4B). In other words, this result shows that cells start to round up 40 to 50 s after cAMP begins to rise.

In order to measure to what extent this event triggers a modification of cell trajectory, we measured the angle formed between the polarization axes observed between two consecutive lamellipodia separated by cAMP-induced lamellipodium retraction. As displayed on Figure 3G, the cells preferentially repolarize with an angle between 90° and 180° (Mean of 108.4 ± 4.7; *N* = 111 repolarization events from 37 cells) showing that cAMP transients favor directional changes.

### Control of Stable Actin Relocalization by cAMP

**FIGURE 5:**
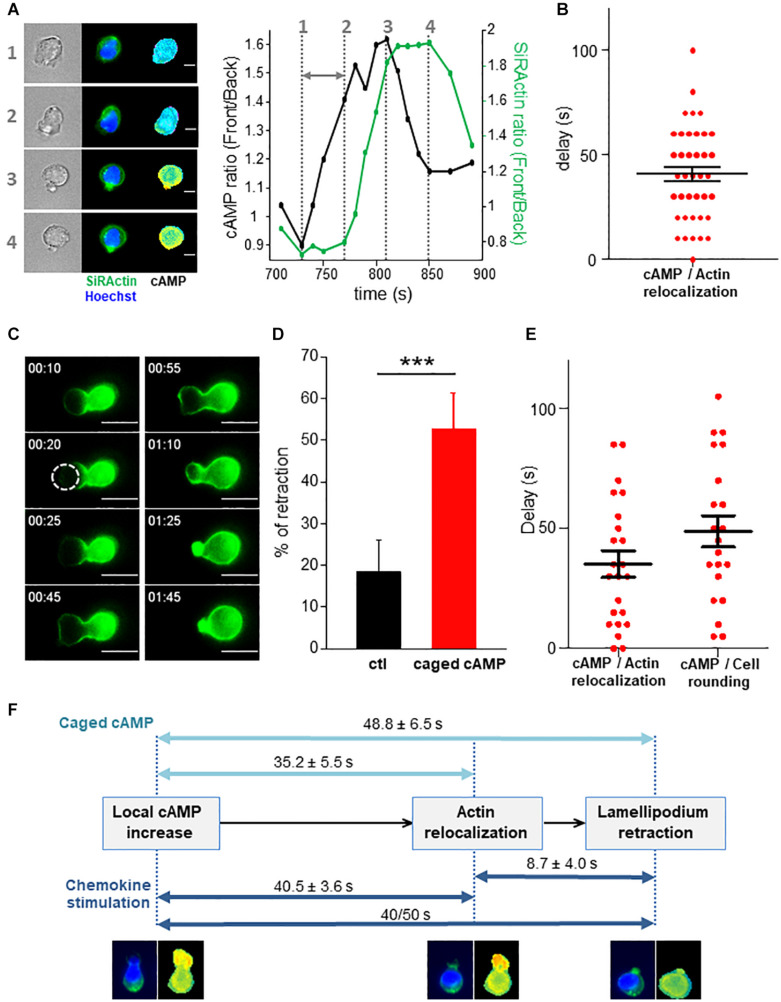
cAMP increase drives stable actin relocalization. **(A)** Example of stable actin distribution recorded simultaneously with cAMP variations. The quantification of the two events is shown on the graph and the delay between the two is indicated by the gray arrow. The numbers show the times of the 4 steps. The complete series of images is shown in Supplementary Movie 7. Scale bar = 10 μm. **(B)** Delays between the beginning of cAMP increase and SiRActin relocalization measured as shown in (A). Values correspond to the mean ± SE of delays corresponding to 39 retraction events from 21 different cells. **(C)** Example of rounding up and actin relocalization induced by local release of cAMP after illumination at 405 nm. The cell was incubated previously with DMACM-caged 8-Br-cAMP and labeled with SiRActin. The size of the laser spot is indicated by the white circle. The complete series of images is shown in Supplementary Movie 8. Scale bar = 10 μm. **(D)** For each experiment, the frequency of lamellipodium retraction taking place within 2 min after illumination was quantified in cells pre-incubated with DMSO (ctl) or caged cAMP. Values correspond to the mean of nine independent experiments (6–21 cells/experiments). ***p < 0.001 paired *t*-test. **(E)** The delay between cAMP release and stable actin relocalization or increase of the roundness value was measured. For each cell, relocalization of SiRActin was measured over time as well as the roundness. The delay corresponds to the moment at which the values start to increase. Values correspond to the mean ± SE of 23 cells for actin relocalization and 21 for the roundness. **(F)** Summary of the three sequential steps leading to cell rounding: local increase in cAMP, recruitment of stable actin at the cell front cell, retraction of the lamellipodium leading to the rounding up of the cells. The delays measured in the different experiments are indicated. Dark blue: observation of migrating cells, light blue: artificial increase of cAMP induced by local photo-release of caged-cAMP.

To address the direct link between cAMP increase and stable actin recruitment, the two parameters were monitored simultaneously. As shown in the example presented in Figure 5A and in Supplementary Movie 7, a local increase of cAMP can first be observed in the lamellipodium which is followed by a recruitment of stable actin at this position. This observation has been quantified over time by measuring the front/back ratio for cAMP along a scanline together with the stable actin recruitment (Figure 5A, right panel) and the time lag was measured (gray arrow). The delay between the two events was 40.5 ± 3.6 s (*N* = 39 retraction events from 21 different cells, Figure 5B). This result indicates that the local increase in cAMP appears first, followed by the recruitment of stable actin. In order to establish with certainty the causal link between the two events, we artificially generated a local increase in cAMP in the lamellipodium by using a caged form of the nucleotide (DMACM-caged 8-Br-cAMP). Unfortunately, this compound is weakly fluorescent and the release of a coumarin analog in addition to 8-Br-cAMP upon DMACM-caged 8-Br-cAMP uncaging, prevents us to measure simultaneously with our FRET biosensor, the rise in cAMP we trigger in these conditions. However, we can expect that the use of this compound allowed us to generate a transient rise in cAMP after illumination at 405 nm as we observed in a previous study (Conche et al., 2009) and to analyze its consequences on the distribution of stable actin, together with cell roundness. Illumination of the leading edge on a 7 μm diameter region induces the recruitment of SiRActin and a lamellipodium retraction when cells have been incubated with caged 8-Br-cAMP (Figure 5C and Supplementary Movie 8), but not in control conditions (Supplementary Movie 9). The frequency of retraction upon laser illumination was significantly higher in cells which had been loaded with caged-cAMP compared to control cells (Figure 5D). The retraction events observed in control cells probably correspond to illumination-induced or spontaneous retraction events. After cAMP-induced retraction, cells remain round or form a new lamellipodium in another direction (Supplementary Movie 8). As shown in Figure 5E, the accumulation of stable actin starts 35.2 ± 5.5 s (*N* = 23 cells) after cAMP release, while the lamellipodium begins to retract after 48.8 ± 6.5 s (*N* = 21 cells). This result demonstrates that a local increase in cAMP is sufficient to induce the recruitment of stable actin and the subsequent retraction of the lamellipodium. Interestingly, by generating an artificial increase in cAMP, the delays measured between the three steps (increase in cAMP/relocalization of stable actin/retraction of the lamellipodium) were very similar to those measured in chemokine-stimulated cells (Figure 5F), suggesting the involvement of a similar sequence of events in the two configurations and pointing out to cAMP as the upstream initial trigger of the whole sequence of cytoskeletal-driven morphological alterations responsible for directional changes in T cell migration.

## Discussion

T cell migration conditions an efficient immune response. The rapid and random displacement of these cells constitutes an important property for an optimization strategy for foreign antigen detection. Although anatomical constraints might impose T cell trajectory, we focus here on the influence of the chemical environment, i.e., chemokine, on T cell migration. We demonstrate that cell intrinsic factors are sufficient to promote random migration upon chemokine stimulation. Our results highlight a three step time sequence of signaling events, summarized in Figure 5F. Altogether, our results demonstrate that during T cell migration, a pool of actin corresponding to actomyosin displays an asymmetrical distribution. Surprisingly, this pool is mobile and sets the cell polarity: while it is restricted to the back of the cell upon migration, it is recruited at the lamellipodium upon cell rounding. We have shown that this redistribution is triggered by a rise in cAMP which starts at the cell front before invading the whole cell. Interestingly, the cAMP-induced lamellipodium retraction is followed by the repolarization of the cells in another direction with a mean angle of about 110° and therefore promotes the exploratory behavior of the cells.

We may wonder what triggers cAMP bursts observed during T cell migration. The cells displaying repetitive elongation/retraction cycles as observed in some of our experiments might be a good model to address this issue. cAMP oscillations indicate that cells are able to synthetize and degrade cAMP at high frequency (2.5 min). Surprisingly, the oscillation period is very similar from cell to cell, which suggests a universal cell-intrinsic cross-talk between adenylate cyclases and phosphodiesterases, the enzymes which, respectively, synthetize and degrade cAMP. One interesting possibility would be that cell deformation by itself, i.e., membrane stretching, could be the driving force of cAMP bursts. Indeed, the increase in membrane tension generated during migration (Pontes et al., 2017), might drive a cAMP increase, as suggested in other systems (Alenghat et al., 2009; McKenzie et al., 2020). In this context, the cAMP-induced recruitment of actomyosin would reduce this stretch by retracting the lamellipodium, and therefore inhibit the synthesis of cAMP. In parallel, cAMP increase *via* protein kinase A, one of the main targets of cAMP (Torres-Quesada et al., 2017), could activate phosphodiesterases (Gancedo, 2013) [such as PDE4 highly expressed in T cells (Sheth et al., 1997)] thus accelerating the cAMP decrease.

The link between cAMP and local recruitment of stable actin is another puzzling observation. As summarized in Figure 5F, a 40–50 s delay is necessary for stable actin to increase at the front after cAMP rise, suggesting that the link between the two events involves a multi-step signaling cascade which might involve PKA. Furthermore, upon lamellipodium retraction, we have observed a restricted zone of stable actin accumulation although the cAMP increase finally invades the whole cell. This suggests that a signal is generated very locally after cAMP increases. An interesting possibility would be the involvement of A Kinase anchoring proteins (AKAP), a family of proteins which would be able to convert the diffusible signal brought by cAMP into spatially restricted PKA activity (Dema et al., 2015).

Finally, the local recruitment of actomyosin at the lamellipodium results in its retraction within few seconds. Upon neutrophil migration, local increases in cAMP observed at the back of the cell have been reported to induce uropod retraction due to the regulation of non-muscle myosin IIA activity by PKA (Liu et al., 2010). Therefore, while cAMP promotes cell migration in neutrophils, it promotes exploratory behavior in T cells. Indeed, the local increase in cAMP is observed at the T cell front and promotes lamellipodium retraction (and then a change of trajectory) rather than at neutrophil back to promote uropod retraction (and therefore favoring migration). Furthermore, in T cells, retraction is due to cAMP-induced actomyosin relocalization rather than an increase in actomyosin activity as observed in neutrophils.

cAMP is generally considered as a messenger which dampens immune responses (Mosenden and Tasken, 2011). However, this statement must be qualified according to the characteristics of the cAMP increase. Indeed, for T cell activation, although high and sustained cAMP rises have been reported to inhibit TCR signaling such as calcium increase, lck activation or IL2 production (Henney and Lichtenstein, 1971; Tamir et al., 1996; Vang et al., 2001; Hermann-Kleiter et al., 2006; Daher et al., 2019), we have previously shown that T cell adhesion to antigen-presenting cells triggers a transient increase in cAMP which lowers the antigen detection threshold and therefore favors T cell responses (Conche et al., 2009). Concerning migration, similarly, high and sustained cAMP rises triggered by pharmacological drugs, PGE2 or β-adrenergic receptors stimulation, are known to inhibit T cell motility (Valitutti et al., 1993; Oppenheimer-Marks et al., 1994; Layseca-Espinosa et al., 2003; Dong et al., 2006). However, our present study highlights on the contrary that transient bursts in cAMP, by remodeling the actin cytoskeleton, might favor the exploratory behavior of T cells, a crucial step to mounting an efficient immune response. It might therefore be important to revisit the immunosuppressive effect of cAMP. Indeed, spatiotemporal and intensity control of cAMP signal is crucial for T cell properties: although sustained rise of cAMP may be inhibitory, transient increase of this messenger may, conversely, favor T cell responses.

## Data Availability Statement

The original contributions presented in the study are included in the article/Supplementary Material, further inquiries can be directed to the corresponding author/s.

## Author Contributions

MS, FR, ST, AF, RT, MF, AB, VF, JD, and CR performed experiments and analyzed data. ML helped with the experimental design of Traction Force Microscopy experiments. AF wrote R code for gel rigidity measurements. MS, JD, and CR designed experiments and wrote the manuscript. All authors contributed to the article and approved the submitted version.

## Conflict of Interest

The authors declare that the research was conducted in the absence of any commercial or financial relationships that could be construed as a potential conflict of interest.
