# Supplementary material for: cAMP Bursts Control T Cell Directionality by Actomyosin Cytoskeleton Remodeling
Source: Front Cell Dev Biol. 2021 May 20;9:633099. doi: 10.3389/fcell.2021.633099 (PMC8173256; doi:10.3389/fcell.2021.633099)
Supplement: Supplementary file 10 [file Data_Sheet_1.docx]

Supplementary Material
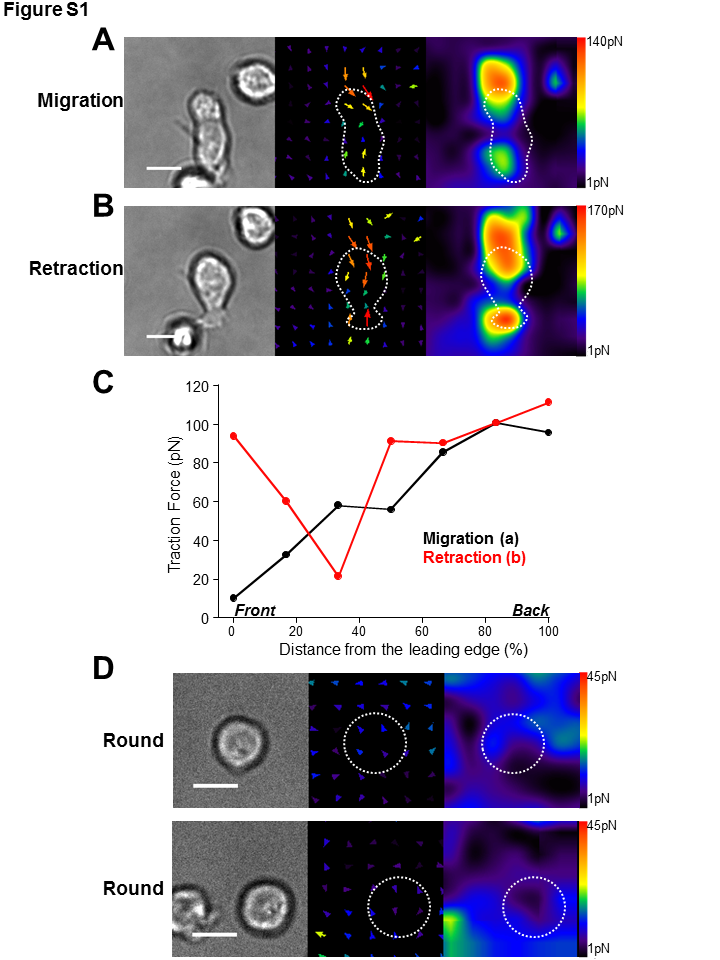


**Figure S1: Example of Traction Force Microscopy**

**A-** CXCL12-stimulated cell migrating (left panel: transmitted light picture) on VCAM-1 coated gel develops centripetal forces (vectorplot, center panel) whose magnitude is at a maximum at the back (Heatmap, right panel). The cell outline is indicated by a white dotted line. Note that the size of the gel area deformed by the cell is larger than the cell itself. The color code indicates the traction force intensity in picoNewton.

**B-** The same cell as in **(A)** is retracting its lamellipodium. Although the forces are still centripetal (vectorplot, center panel), similar intensities can be measured at the back and at the cell front (Heatmap, right panel). As in **(A)** the size of the gel area deformed by the cell is larger than the cell itself. The cell outline is indicated by a white dotted line.

**C-** Quantification of the forces along the cell presented in **(A** & **B)** upon migration and retraction of its lamellipodium.

**D**- Two examples of cells which no longer imprint forces on the gel after lamellipodium retraction. Vectorplot in the center and Heatmap on the right.

Scale bar = 10µm


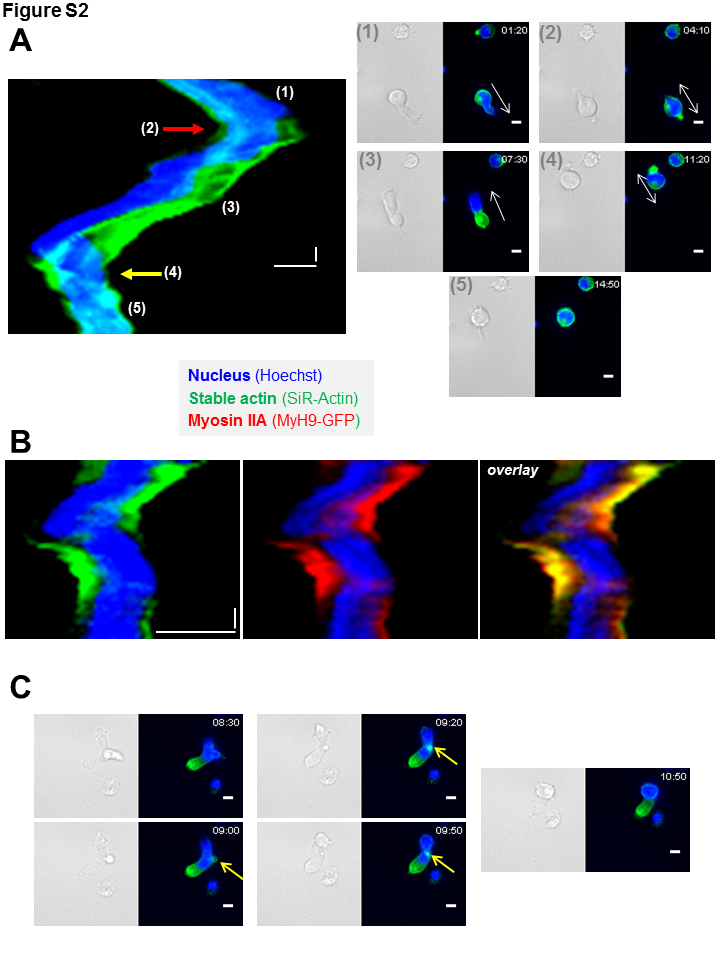


**Figure S2: Stable actin and non-muscle myosin IIA relocalization upon lamellipodium retraction**

**A**- A typical example of stable actin distribution (SiRActin) and nucleus localization (Hoechst) is shown on the kymograph. The x axis corresponds to the average SiRActin intensity along the cell while the y axis corresponds to time. Numbers indicate the different steps: (1) and (3): the cell is migrating. (2), red arrow: it changes its direction. (4), yellow arrow: the cell rounds up. (5): the cell remains round. The associated images are shown on the right panel and the complete series of images in Movie S3. Horizontal scale bar = 10µm, vertical scale bar = 1min.

**B**- A typical example of stable actin (SiRActin), Myosin IIA (MyH9-GFP) distributions and nucleus localization (Hoechst) is shown on the kymographs. The x axis corresponds to the average SiRActin, MyH9-GFP or Hoechst intensities along the cell while the y axis corresponds to time. The complete series of images is shown in Movie S4. Horizontal scale bar = 10µm, vertical scale bar = 1min.

**C**- Example of a cell developing two simultaneous lamellipodia. The local recruitment of stable actin (yellow arrow) allows the retraction of one of them.

**Figure S3**


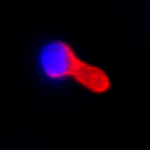

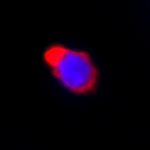

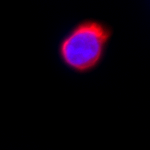


**Migration**

**Retraction**

**Rounding**

0

1

2

3

4

5

Utrophin ratio (Front/Back)

ns

***

***

**A**

**B**

**Figure S3: Utrophin relocalization upon lamellipodium retraction**

A- In cells transfected with the Utr-CH-RFP construct, the front to back ratio intensities were measured by drawing a scanline along the axis of cells upon migration, while the lamellipodium retracted and once the cell had rounded up. Values correspond to the mean ± SE of 39 events from 32 different cells (migration), 30 events from 23 different cells (lamellipodium retraction) and 5 rounding events from 5 different cells (after retraction). Statistical analysis was performed through a 1way ANOVA test with a Tukey post-test. **** p<0.0001.

B- Example of Utrophin (in red) distribution during migration, lamellipodium retraction and in round cells. Nucleus is labeled with DAPI (in blue).


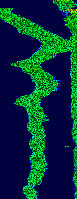

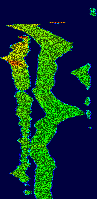


low

high

**Ca**

(ratio)


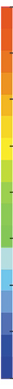


ns

ns

ns

**Migration**

**Retraction**

**Round**

0.0

0.5

1.0

1.5

2.0

Ca ratio (Front/Back)

**A**

**B**

**C**


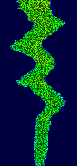

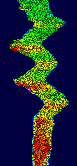


**cAMP**

**Ca**


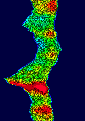

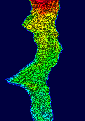


**cAMP**

**Ca**

**Figure S4**

**Cell 1**

**Cell 2**

**Figure S4: Calcium levels during migration**

**A**- Kymographs of two examples of calcium measurements in Fura-2-loaded T cells deposited on CXCL12/VCAM-1 coated coverslips. No variation in Calcium could be detected. Calcium level is coded in false color. Horizontal scale bar = 10µm, vertical scale bar = 1min.

**B**- Ratios between the front and the back levels of Calcium have been measured by drawing scanlines along the cell axis in cells migrating, retracting their lamellipodium or rounding up (after retraction). 57-59 values ± SE from 14 different cells.

**C**- Kymographs of two examples of simultaneous cAMP (TEpacVV) and Calcium (Fura-2) recordings. Although some cAMP increase could be observed when cells changed direction, no calcium variations appeared. Horizontal scale bar = 10µm, vertical scale bar = 1min.

1

1.1

1.2

1.3

1.4

1

1.1

1.2

1.3

1.4

1.5

0

200

400

600

800

1000

**Cell 1**

cAMP (R/R0)

**Cell 2**

cAMP (R/R0)

time (s)

**A**

**B**

0

100

200

300

400

500

Oscillation period (s)

0

10

20

30

40

Standard error (s)

**C**

**Figure S5**

**Figure S5: cAMP oscillations upon elongation/retraction cycles**

**A**- Example of two different TEpacVV-transfected cells displaying cAMP oscillations. cAMP values have been normalized to the initial ratio (R0).

**B**- Distribution of cAMP oscillating periods. The mean ± SE corresponds to the average period measured in 29 cells displaying 3-14 oscillations. Each dot represents one cell. Note the low dispersion of the period among the different cells.

**C**- For each cell displaying 3-14 cAMP oscillations, the delay between 2 cAMP peaks was measured and the average period calculated. The regularity of the oscillations was assessed by calculating the standard error around their average. The small dispersion of the values indicates that, for a given cell, the oscillations are very regular.

**Movie S1: Random migration on VCAM-1 / CXCL12 coated coverslip**

**
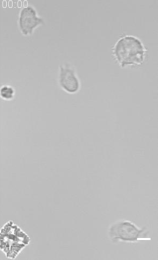
**Transmitted light images of T lymphocytes migrating randomly. Scale bar = 10µm.

**
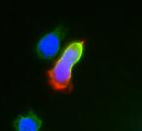
Movie S2:** **Asymmetry of actin networks upon chemokine-stimulated T cells**

Example of a T cell transfected with LifeAct-mCherry and labelled with SiRActin and Hoechst deposited on a VCAM-1/CXCL12-coated coverslip and observed. Image analysis of this cell in Fig 1A. Scale bar = 10µm.

**Movie S3: Stable actin relocalization**

**
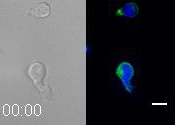
**Example of a T lymphocyte labelled with SiRActin and Hoechst deposited on a VCAM-1/CXCL12-coated coverslip and monitored upon migration. The corresponding kymograph is shown in Fig S2A. Scale bar = 10µm.

**Movie S4: Non-muscle Myosin IIA relocalization**

**
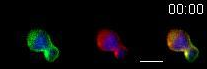
**Example of a T lymphocyte transfected with MyH9-GFP and labelled with SiRActin and Hoechst deposited on a VCAM-1/CXCL12-coated coverslip and monitored upon migration. The corresponding kymograph is shown in Fig S2B. Scale bar = 10µm.

**Movie S5:** **cAMP variations upon migration**

**
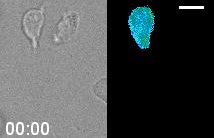
**TEpacVV-transfected T cell migrating on a CXCL12/VCAM-1-coated coverslip. cAMP is coded in false colors. The corresponding kymograph is shown in Fig 3A. Scale bar = 10µm.

**
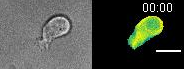
Movie S6:** **cAMP oscillations upon elongation/retraction cycles**

TEpacVV-transfected T cell migrating on a CXCL12/VCAM-1 coated coverslip. cAMP is coded in false colors. The corresponding kymograph is shown in Fig 3D. Scale bar = 10µm.

**Movie S7: cAMP increase and stable actin relocalization**

**
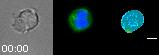
**TEpacVV-transfected T cell loaded with SiRActin deposited on a CXCL12/VCAM-1-coated coverslip. Detailed images in Fig 5A. cAMP is coded in false colors. Scale bar = 10µm.

**Movie S8: Effect of caged-cAMP photo-release on stable actin relocalization and cell rounding**


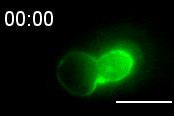
The cell was incubated previously in DMACM-caged 8-Br-cAMP and labeled with SiRActin. At 20s, it was illuminated at 405nm on a spot of 7µm diameter (white circle). Detailed images in Fig 4C. Scale bar = 10µm.


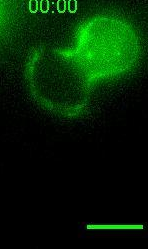
 **Movie S9: Effect of laser illumination on stable actin distribution**

The cell was incubated previously in DMSO (1/500) and labeled with SiRActin. At 20s, it was illuminated at 405nm on a spot of 7µm diameter (white circle). Scale bar = 10µm.
